# Is There any Association Between Celiac Disease and *Helicobacter pylori?*

**DOI:** 10.5005/jp-journals-10018-1179

**Published:** 2016-12-01

**Authors:** Ahmet Uyanikoglu, Huseyin Dursun, Necati Yenice

**Affiliations:** 1 Department of Gastroenterology, Harran University, Sanliurfa, Turkey; 2 Department of Internal Medicine, Harran University, Sanliurfa, Turkey

**Keywords:** Celiac disease, *Helicobacter pylori*, Prevalence.

## Abstract

**Objective:**

It was aimed to determine if there was a correlation between celiac disease (CD) and *Helicobacter pylori (H. pylori)* by comparing the prevalence of *H. pylori* in patients with and without CD.

**Materials and methods:**

The patients who were diagnosed with CD and tested for *H. pylori* and the patients who presented for gastroscopy and tested for *H. pylori* were evaluated retrospectively and the prevalence of *H. pylori* was compared.

**Results:**

Fifteen (48%) of 31 patients who were diagnosed with CD and tested for *H. pylori* were males and had a mean age of 33.1 ± 12.7 years (17–72). In the CD group, *H. pylori* was positive in 15 patients (48%), and 592 antrum biopsy that was performed were included as the control group. Of these patients, 299 (50.5%) were males and had a mean age of 44.4 ± 17.05 years (16–96). *Helicobacter pylori* were positive in 316 patients (53.4%). *Helicobacter pylori* prevalence was similar in the groups with and without CD (p > 0.5).

**Conclusion:**

Although the prevalence of *H. pylori* was lower in celiac patients compared to the control group, the difference was not statistically significant. Although no findings suggesting a correlation between CD and *H. pylori* was found, further studies should be conducted.

**How to cite this article:**

Uyanikoglu A, Dursun H, Yenice N. Is There any Association Between Celiac Disease and *Helicobacter pylori*? Euroasian J Hepato-Gastroenterol 2016;6(2):103-105.

## INTRODUCTION

The etiopathology of celiac disease (CD) is not known very well and varying prevalence of *Helicobacter pylori* (*H. pylori*) in these patients have been reported in the literature. In a large series from USA, CD was found in 2,689 (2%) of 136,179 patients. Among these patients, the prevalence of *H. pylori* was found to be markedly lower in the ones with CD (4.4%) compared to the ones who did not have CD (8.8%).^1^ In another study, similarly, the prevalence of *H. pylori* and peptic ulcer was found to be markedly lower in the patients who had CD compared to the control group.^2^

In contrast to the studies in which the prevalence of *H. pylori* was found to be lower in celiac patients, the prevalence of *H. pylori* was reported not to be increased in children with CD in another study.^3^ In a study conducted in Turkey, *H. pylori* was found to be positive in 21 patients with CD (21.8%) and in 56 patients (23.8%) in the control group and no difference was found between the two groups.^4^

In this study, it was aimed to determine if there was a correlation between CD and *H. pylori* by comparing the prevalence of *H. pylori* in patients with and without CD.

## MATERIALS AND METHODS

The patients who were diagnosed with CD and tested for *H. pylori* between December 2011 and September 2014 and the patients who presented to our endoscopy unit for gastroscopy and tested for *H. pylori* between December 2011 and November 2012 were evaluated retrospectively and the prevalence of *H. pylori* was compared.

In both groups, antrum biopsy samples were stained with hematoxylin-eosin and modified Giemsa method after appropriate preparation and *H. pylori* was investigated using a light microscope.

The Ethics Committee approval was not received because the study was retrospective.

## RESULTS

Fifteen (48%) of 31 patients whose gastroscopy was performed and who were diagnosed with CD and tested for *H. pylori* were males and had a mean age of 33.1 ± 12.7 years (17–72). In the CD group, *H. pylori* was positive in 15 patients (48%) and negative in 16 patients (52%). The most common finding accompanying CD was antral gastritis, which was found in 25 patients (80%).

In addition, 841 patients were screened retrospectively and 592 of these patients, whose antrum biopsies were obtained, were included as the control group. Of these patients, 299 (505%) were males and had a mean age of 44.4 ± 17.05 years (16–96). *Helicobacter pylori* was positive in 316 patients (53.4%) and negative in 276 patients (46.6%).

In comparison of the two groups, the CD group was younger (p < 0.05). Gender distribution was similar in the patients with and without CD (p > 0.5). Although *H. pylori* positivity was found with a lower rate in the CD group, there was no statistically significant difference compared to the patients who did not have CD (p > 0.5). The results are summarized in Table 1.

**Table 1:** **Table 1:** Prevalence of *H. pylori* in celiac diseases

		*Celiac patients*		*Control*		*p-value*	
Age (years)		33.1 ± 12.7 (17–72)		44.4 ± 17.05 (16–96)		< 0.05	
Gender (male)		15 (48%)		299 (50.5%)		> 0.05	
*H. pylori* positive		15 (48%)		316 (53.4%)		> 0.05	
*H. pylori* negative		16 (52%)		276 (46.6%)		> 0.05	

## DISCUSSION

The prevalence of CD has increased in the last decade, the reason of which is not known well. According to the hygiene hypothesis, a decrease in exposure to bacterial antigens may trigger autoimmunity. It is controversial if there is a correlation between *H. pylori* infection and CD. In a study in which patients who cross-sectionally underwent gastroscopy and gastric and duodenal biopsies were evaluated, an inverse correlation was found between CD and presence of *H. pylori*. The frequency of *H. pylori* was found to be markedly lower (4.4%) in the subjects who had CD compared to the ones who did not have CD (8.8%; p < 0.0001). It was recommended to investigate if *H. pylori* affected the immune response to intake of gluten.^1^ The prevalence of *H. pylori* infection and peptic ulcer is markedly lower in patients with CD compared to controls. It has been reported that changes triggered in the intestinal and/or host immune response by gluten-free diet might explain increased prevalence of *H. pylori* in patients who are treated.^2^ In our study, *H. pylori* was found with a lower rate in the subjects who had CD similar to these studies, but the difference was not statistically significant. This may be related with the low number of patients.

In a study in which the correlation between gastritis and CD was investigated, superficial gastritis was the most common finding in CD in children and adolescents. This study suggested that occurrence of lymphocytic gastritis might be related with long-term exposure to gluten.^5^ In an Iranian study including 250 patients, it was investigated if CD was related with gastric anomalies. It was concluded that the clinical presentation of CD was not different in the subjects with *H. pylori* infection and histology was nonspecific and unhelpful even if the patient had positive serology. *H. pylori* infection and chronic gastritis were found with a high rate and no relation with CD was found.^6^ In our study, the most common additional gastroscopic finding was antral gastritis in the patients who had CD. Similar to the Iranian study, we found the prevalence of *H. pylori* infection and chronic gastritis to be markedly higher in both groups with and without CD compared to the western studies.

Histopathological examination is still the gold standard for the diagnosis of CD, gastric lesions, and *H. pylori* infection.^7^ We also based our diagnoses of CD and *H. pylori* infection on histopathological examination.

Celiac disease may be related with *H. pylori* gastritis, but does not affect the clinical presentation except for abdominal distention; CD is related with mild duodenal lesions. Gluten-free diet provides improvement in the symptoms in all patients independent of *H. pylori* gastritis. In presence of *H. pylori* gastritis, gastric metaplasia increases. It has been reported that further prospective clinical and histopathological investigations related with *H. pylori*-related gastric metaplasia in patients with CD are needed.^4^ In our study, we did not assess the clinical findings and gastric metaplasia.

In patients with *H. pylori* gastritis, duodenal intra­epithelial lymphocytes seem to be increased. This can be reversed with eradication of *H. pylori*. In one study, it was investigated if CD patients infected with *H. pylori* showed different clinicopathological properties compared to noninfected patients and if the histopathological responses to gluten-free diet were different in infected and noninfected patients. The clinical properties in CD patients were not related with *H. pylori* gastritis and the response to gluten-free diet was similar in both patient groups. The increased prevalence in mild duodenal lesions and lymphocytosis triggered by *H. pylori* gastritis in CD patients infected with *H. pylori* was found to cause deep inflammation and stricture changes with a lower rate compared to mucosal changes. This study provided further support to the pathogenetic relation between CD and lymphocytic gastritis.^8^ It was stated that increased intraepithelial lymphocytes in some cases might be related with inappropriate host response to *H. pylori,* and screening for *H. pylori* and eradication of *H. pylori* should be considered before gluten-free diet.^9^ All these data and our study suggest that *H. pylori* should be investigated in patients with CD.

Conclusively, the prevalence of *H. pylori* examined on antrum biopsies of celiac patients and on patients who underwent routine gastroscopy procedure were found to be lower in the celiac group, but the difference was not statistically significant. In both groups, *H. pylori* is positive in approximately half of the patients. Further studies, including a higher number of patients, are needed to determine the correlation between CD and *H. pylori*.

## References

[1] Lebwohl B, Blaser MJ, Ludvigsson JF, Green PH, Rundle A, Sonnenberg A, Genta RM (2013). Decreased risk of celiac disease in patients with *Helicobacter pylori* colonization.. Am J Epidemiol.

[2] Ciacci C, Squillante A, Rendina D, Limauro S, Bencivenga C, Labanca F, Romano R, Mazzacca G (2000). *Helicobacter pylori* infection and peptic disease in coeliac disease.. Eur J Gastroenterol Hepatol.

[3] Luzza F, Mancuso M, Imeneo M, Mesuraca L, Contaldo A, Giancotti L, La Vecchia AM, Docimo C, Pensabene L, Strisciuglio P, et al. (1999). *Helicobacter pylori* infection in children with celiac disease: prevalence and clinicopathologic features.. J Pediatr Gastroenterol Nutr.

[4] Aydogdu S, Cakir M, Yuksekkaya HA, Tumgor G, Baran M, Arikan C, Yagci RV (2008). *Helicobacter pylori* infection in children with celiac disease.. Scand J Gastroenterol.

[5] Nenna R, Magliocca FM, Tiberti C, Mastrogiorgio G, Petrarca L, Mennini M, Lucantoni F, Luparia RP, Bonamico M (2012). Endoscopic and histological gastric lesions in children with celiac disease: mucosal involvement is not only confined to the duodenum.. J Pediatr Gastroenterol Nutr.

[6] Vives MJ, Esteve M, Marine M, Fernández-Bañares F, Alsina M, Salas A, Loras C, Carrasco A, Almagro P, Viver JM, et al. (2012). Prevalence and clinical relevance of enteropathy associated with systemic autoimmune diseases.. Dig Liver Dis.

[7] Ghimpu S, Bozomitu L, Cardei E, Oltean C, Burlacu M, Anton D, Trandafir L, Mihăilă D, Moraru D (2009). *Helicobacter pylori* infection in children with celiac disease.. Rev Med Chir Soc Med Nat Lasi.

[8] Villanacci V, Bassotti G, Liserre B, Lanzini A, Lanzarotto F, Genta RM (2006). *Helicobacter pylori* infection in patients with celiac disease.. Am J Gastroenterol.

[9] Nahon S, Patey-Mariaud De Serre N, Lejeune O, Huchet FX, Lahmek P, Lesgourgues B, Traissac L, Bodiguel V, Adotti F, Tuszynski T, et al. (2006). Duodenal intraepithelial lymphocytosis during *Helicobacter pylori* infection is reduced by antibiotic treatment.. Histopathology.

